# Normalization method for metabolomics data using optimal selection of multiple internal standards

**DOI:** 10.1186/1471-2105-8-93

**Published:** 2007-03-15

**Authors:** Marko Sysi-Aho, Mikko Katajamaa, Laxman Yetukuri, Matej Orešič

**Affiliations:** 1 VTT Technical Research Centre of Finland, Tietotie 2, P.O. Box 1500, FIN-02044 VTT, Espoo, Finland; 2 Turku Centre for Biotechnology, Tykistökatu 6, FIN-20521, Turku, Finland

## Abstract

**Background:**

Success of metabolomics as the phenotyping platform largely depends on its ability to detect various sources of biological variability. Removal of platform-specific sources of variability such as systematic error is therefore one of the foremost priorities in data preprocessing. However, chemical diversity of molecular species included in typical metabolic profiling experiments leads to different responses to variations in experimental conditions, making normalization a very demanding task.

**Results:**

With the aim to remove unwanted systematic variation, we present an approach that utilizes variability information from multiple internal standard compounds to find optimal normalization factor for each individual molecular species detected by metabolomics approach (NOMIS). We demonstrate the method on mouse liver lipidomic profiles using Ultra Performance Liquid Chromatography coupled to high resolution mass spectrometry, and compare its performance to two commonly utilized normalization methods: normalization by *l*_2 _norm and by retention time region specific standard compound profiles. The NOMIS method proved superior in its ability to reduce the effect of systematic error across the full spectrum of metabolite peaks. We also demonstrate that the method can be used to select best combinations of standard compounds for normalization.

**Conclusion:**

Depending on experiment design and biological matrix, the NOMIS method is applicable either as a one-step normalization method or as a two-step method where the normalization parameters, influenced by variabilities of internal standard compounds and their correlation to metabolites, are first calculated from a study conducted in repeatability conditions. The method can also be used in analytical development of metabolomics methods by helping to select best combinations of standard compounds for a particular biological matrix and analytical platform.

## Background

Metabolomics is a discipline dedicated to the global study of metabolites, their dynamics, composition, interactions, and responses to interventions or to changes in their environment, in cells, tissues, and biofluids. Concentration changes of specific groups of metabolites may be descriptive of systems responses to environmental or genetic interventions, and their study may therefore be a powerful tool for characterization of complex phenotypes [1-3] as well as for development of biomarkers for specific physiological responses [4,5].

Study of the variability of metabolites in different states of biological systems is therefore an important task of systems biology. As we are primarily interested in systems responses resulting in metabolite level regulation as related to diverse genetic or environmental changes, it is important to separate such *interesting *biological variation from *obscuring *sources of variability introduced in experimental studies of metabolites. Since multiple experimental platforms are commonly applied in the studies of metabolites [6,7], the sources of the obscuring variation are many and platform specific [8]. Such sources include variability arising from inhomogeneity of samples, their lability and inevitable minor differences in sample preparation. In mass spectrometry based detection, the sources include the variations in the ion source as well as matrix specific effects such as ion suppression [9]. Following the measurement, the data preprocessing steps such as peak detection, peak integration and alignment may introduce an additional error.

Chemical diversity of metabolites, leading for example to different recoveries during extraction or responses during ionization in mass spectrometer, makes separation of interesting and obscuring variation a difficult task. Quantitative analytical methods have commonly relied on utilization of isotope labeled internal standard for each metabolite measured. However, in broad metabolic profiling approaches this is not practical. The number of metabolites is large, they are chemically too diverse to afford a common labeling approach, and many of them may not even be known. The availability of stable isotope labeled references is generally also very limited.

Strategies for normalization of metabolic profile data can be divided into two major categories:

• Statistical models used to derive optimal scaling factors for each sample based on complete dataset [10], such as normalization by unit norm [11] or median [12] of intensities, or the maximum likelihood method [2] adopted from the approach developed for gene expression data [13].

• Normalization by a single or multiple internal or external standard compounds based on empirical rules, such as specific regions of retention time [14].

**Figure 1:**
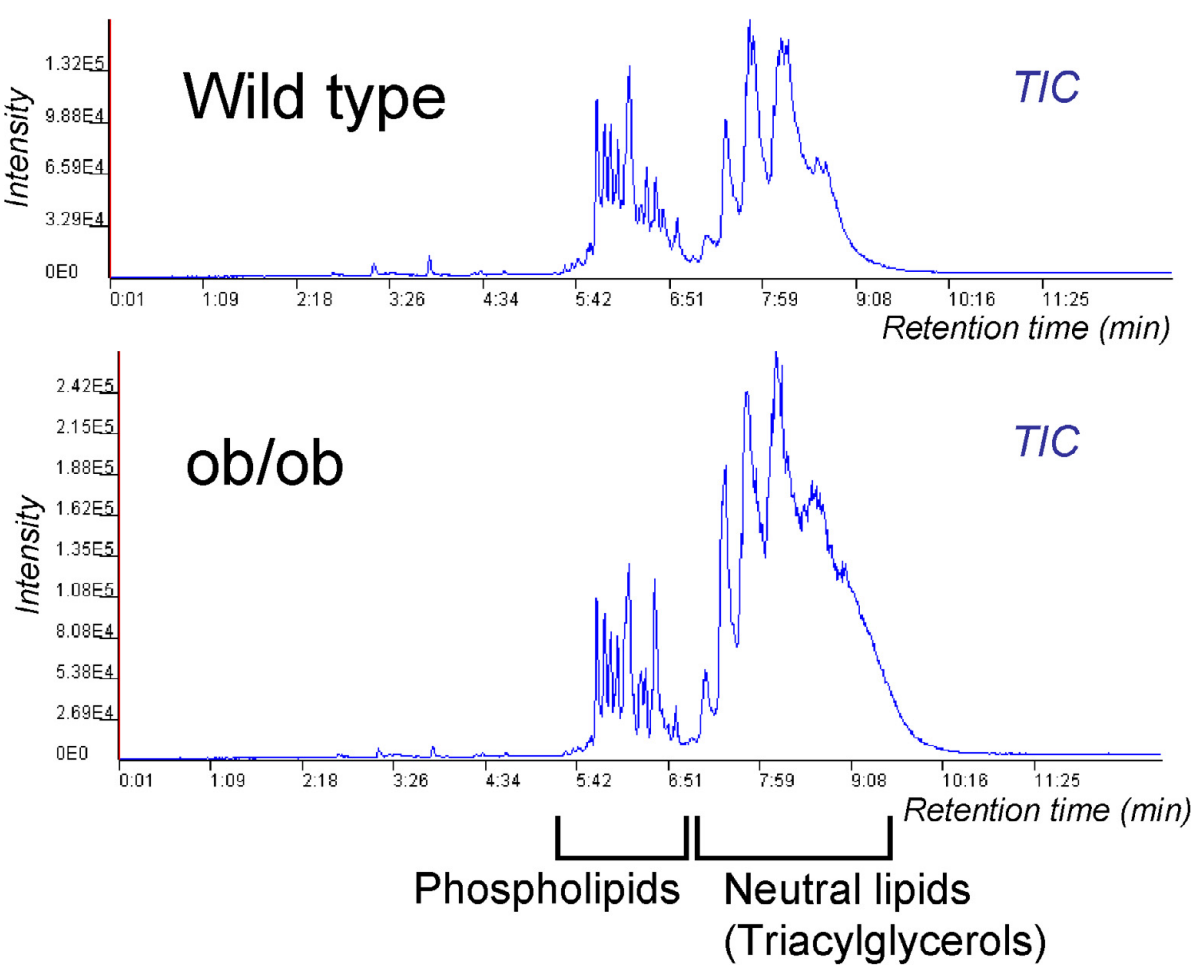
**Comparing two metabolomic total ion chromatograms (TIC) from two different mouse phenotypes**. An illustrative example of how normalization of metabolomics data based on total signal may generate bias in the data. Two mouse liver lipid profiles are drawn to scale, one from obese ob/ob mouse and one from lean wild type mouse. While the phospholipid profiles are similar in total amount, there is a large increase in triacylglycerols in obese mouse. Normalization based on total signal would wrongfully decrease the levels of phospholipids in obese mouse relative to the wild type to balance the increased amount of triacylglycerols.

The statistical approach suffers from the lack of an absolute concentration reference for different metabolites. In addition, constraining the data to a specific norm based on total signal affects its covariance structure, therefore requiring special caution when pursuing multivariate analysis of such data [15]. Metabolites as physiological end-points, largely affected by the environment, do not posses the *self-averaging *property, i.e. concentration increase in a specific group of metabolites is generally not balanced by a decrease of another group. The Figure 1 illustrates this statement. Two total ion chromatograms from Ultra Performance Liquid Chromatography coupled to Mass Spectrometry (UPLC/MS) lipidomics profiling of two different mouse liver samples are shown, one from an obese ob/ob mouse model and one from a lean wild type mouse. The ob/ob mouse model was developed by spontaneous mutation in *ob *gene resulting in lack of leptin [16] and is commonly studied as a model for early onset of severe obesity. Both types of mice have similar levels of phospholipids, but the amount of storage fat in the form of triacylglycerols is markedly increased in the obese mouse. If one would normalize this data based on total signal, such approach would lead to a conclusion that the phospholipids are decreased in the obese mouse (wrong conclusion), while the triacylglycerols are slightly increased (correct qualitatively, but not quantitatively). More sophisticated approaches to normalize metabolomics data based on full profile data have been adopted [2], but the fundamental problem as described above remains.

The choice of multiple internal (added to sample prior to extraction) and external (added to sample after extraction) standard compounds may be a more reasonable choice, but even in that case the *assignment *of the standards to normalize specific peaks remains unclear. One possible approach is to assign a specific standard to metabolite peaks based on similarity in specific chemical property such as retention time in liquid chromatography (LC) column. For example, Bijlsma and colleagues utilize three external standard references for lipid profiling, chosen as mono-, di-, and tri-acyl lipid species representing most abundant lipid classes in their respective region of retention time [14]. Such approach still suffers from at least two problems:

• The retention time is not necessarily descriptive of all matrix and chemical properties leading to obscuring variation. For example, in the lipid separation based on reverse phase LC diverse lipid species such as ceramides, sphingomyelins, diacylglycerols, and several phospholipid classes, are overlapping in retention time, and it is not reasonable to assume same normalization factor can be applied to all these species. The situation is even more complex when analyzing water soluble metabolites.

• The normalization by a single molecular component is very sensitive to its own obscuring variation, which becomes a problem in very complex samples where matrix specific effects such as ion suppression may play an important role.

Recently we introduced a related approach for liquid chromatography – mass spectrometry (LC/MS) that normalizes metabolites based on multiple internal standards, with the normalization factor based on distance to the metabolite peaks both in the retention time and mass-to-charge ratio (m/z) [17]. While such method partially resolves the second issue listed above, it still suffers from the *ad hoc *assignments of internal standard(s) for each component based on a subset of relevant chemical properties.

None of the normalization methods mentioned above systematically take advantage of the obscuring variability that can be learned from the measured data itself. For example, monitoring multiple standard compounds across multiple sample runs may help determine how the standards are correlated, which variation is specific to a particular standard, and which patterns of variation are shared between the measured metabolites and the standards so they can be removed. In this paper we present a new approach to normalization of metabolomics data aiming to address these issues and develop a mathematical model that optimally assigns normalization factors for each metabolite measured based on internal standard profiles. We demonstrate the approach on mouse liver lipid profiling using UPLC/MS, and compare its performance to two other commonly utilized approaches: normalization by *l*_2 _vector norm and by retention time region specific standard compounds. We discuss method performance and several application possibilities.

## Results and discussion

### Formulation of the normalization model

The unnormalized metabolomics data resulting from first stages of preprocessing, usually including peak detection and alignment [17], can be represented by a matrix of *N *variables (metabolite peaks) and *M *objects (samples). For example, in liquid chromatography mass spectrometry (LC/MS) based profiling; each peak is represented by mass to charge ratio (m/z) and retention time (rt).

In the rest of the text we will use the following notation:

• *i *parameterizes peaks: *i *→ {m/z, rt} and *i *= 1...*N*.

• *s *parameterizes peaks from internal standard compounds: *s *→ {m/z, rt} and *s *= 1...*S*

• *j *parameterizes experiment runs: *j *= 1...*M*.

• **X **is *N *by *M *intensity matrix of metabolite peak profiles, with elements *X*_*ij*_

• **Z **is *S *by *M *intensity matrix of standard compound peak profiles, with elements *Z*_*sj*_.

Variation arising from the above mentioned sources is often intensity (or metabolite concentration) dependent, larger variation being associated with higher intensities. The property that the magnitude of variation is not constant is commonly referred to as heteroscedasticity. Therefore, it is reasonable to assume a multiplicative model for the observed intensities:

*X*_*ij *_= *m*_*i *_× *r*_*ij *_(**Z**) × *e*_*ij*_,     (1)

where *m*_*i *_the intensity independent of the run (i.e. true intensity value), *r*_*ij *_(function of **Z**) is the correction factor, and *e*_*ij *_the random error. We assume that the true intensity value depends only on index *i*. In practice this assumption means that we independently measure several samples from one biological specimen (e.g. under repeatability conditions). This assumption is crucial when the normalization model is *trained, i.e*., when the parameters of the model are learned from the data, but it can be relaxed when the normalization is applied to a new set of data. Reasons for this will become clear below.

**Figure 2:**
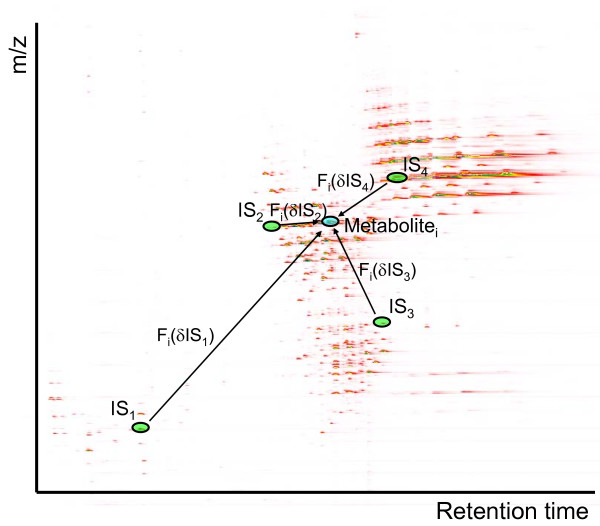
**Illustration of the basic principle of the normalization method**. The normalization factor for each metabolite peak is influenced by the variability of each internal (IS) or external standard component and its association with the variability of the metabolite.

The basic premise of our approach, abbreviated as *NOMIS *method (*N*ormalizatio*n *using *O*ptimal selection of *M*ultiple *I*nternal *S*tandards), is that the systematic variation in measured intensities *X*_ij _for an individual peak *i *can be modeled as a function of variation of standard compounds (Figure 2). Based on this assumption, the correction factors *r*_*ij *_can be determined from the profiles of standard compounds. We log transform the mulitiplicative model of Equation (1)

**Y **= log **X**,** Ω **= log **Z**, **μ **= log **m**, **ρ**(**Ω**) = log **r **(**Z**), **ε **= log **e **    (2)

to obtain an additive model:

*Y*_*ij *_= *μ*_*i *_+ *ρ*_*ij*_(**Ω**) + *ε*_*ij*_.     (3)

The randomness in the values of **Y **is modeled by the error *ρ*_*ij *_that is drawn from the normal distribution with zero mean and variance σi2
 MathType@MTEF@5@5@+=feaafiart1ev1aaatCvAUfKttLearuWrP9MDH5MBPbIqV92AaeXatLxBI9gBaebbnrfifHhDYfgasaacH8akY=wiFfYdH8Gipec8Eeeu0xXdbba9frFj0=OqFfea0dXdd9vqai=hGuQ8kuc9pgc9s8qqaq=dirpe0xb9q8qiLsFr0=vr0=vr0dc8meaabaqaciaacaGaaeqabaqabeGadaaakeaaiiGacqWFdpWCdaqhaaWcbaGaemyAaKgabaGaeGOmaidaaaaa@30F0@:

*ε*_*ij *_~ *N*(0, σi2
 MathType@MTEF@5@5@+=feaafiart1ev1aaatCvAUfKttLearuWrP9MDH5MBPbIqV92AaeXatLxBI9gBaebbnrfifHhDYfgasaacH8akY=wiFfYdH8Gipec8Eeeu0xXdbba9frFj0=OqFfea0dXdd9vqai=hGuQ8kuc9pgc9s8qqaq=dirpe0xb9q8qiLsFr0=vr0=vr0dc8meaabaqaciaacaGaaeqabaqabeGadaaakeaaiiGacqWFdpWCdaqhaaWcbaGaemyAaKgabaGaeGOmaidaaaaa@30F0@).     (4)

We aim at removing the effect of *ρ*_ij _in Equation (3) that we presume to represent such variation in the data that can be explained with changes in the levels of the standard compounds. For the sake of simplicity, we treat the observed values of the standards **Ω **as explanatory variables without modeling their error. We parameterize **ρ **as a linear function of the levels of internal standards:

ρij=∑sβis(Ωsj−〈Ωs.〉) ,     (5)
 MathType@MTEF@5@5@+=feaafiart1ev1aaatCvAUfKttLearuWrP9MDH5MBPbIqV92AaeXatLxBI9gBaebbnrfifHhDYfgasaacH8akY=wiFfYdH8Gipec8Eeeu0xXdbba9frFj0=OqFfea0dXdd9vqai=hGuQ8kuc9pgc9s8qqaq=dirpe0xb9q8qiLsFr0=vr0=vr0dc8meaabaqaciaacaGaaeqabaqabeGadaaakeaaiiGacqWFbpGCdaWgaaWcbaGaemyAaKMaemOAaOgabeaakiabg2da9maaqafabaGae8NSdi2aaSbaaSqaaiabdMgaPjabdohaZbqabaGcdaqadaqaaiabfM6axnaaBaaaleaacqWGZbWCcqWGQbGAaeqaaOGaeyOeI0YaaaWaaeaacqqHPoWvdaWgaaWcbaGaem4CamNaeiOla4cabeaaaOGaayzkJiaawQYiaaGaayjkaiaawMcaaaWcbaGaem4CamhabeqdcqGHris5aOGaeeiiaaIaeiilaWIaaCzcaiaaxMaadaqadaqaaiabiwda1aGaayjkaiaawMcaaaaa@4CF8@

where the average 〈 〉 is taken over the samples *j *= 1...*M*, *i.e*. 〈Ωs.〉≡1M∑jΩsj
 MathType@MTEF@5@5@+=feaafiart1ev1aaatCvAUfKttLearuWrP9MDH5MBPbIqV92AaeXatLxBI9gBaebbnrfifHhDYfgasaacH8akY=wiFfYdH8Gipec8Eeeu0xXdbba9frFj0=OqFfea0dXdd9vqai=hGuQ8kuc9pgc9s8qqaq=dirpe0xb9q8qiLsFr0=vr0=vr0dc8meaabaqaciaacaGaaeqabaqabeGadaaakeaadaaadaqaaiabfM6axnaaBaaaleaacqWGZbWCcqGGUaGlaeqaaaGccaGLPmIaayPkJaGaeyyyIO7aaSaaaeaacqaIXaqmaeaacqWGnbqtaaWaaabeaeaacqqHPoWvdaWgaaWcbaGaem4CamNaemOAaOgabeaaaeaacqWGQbGAaeqaniabggHiLdaaaa@3E3A@. The parameters **β **therefore relate the variability of internal standard intensities with the variability of intensities of endogenous metabolite peaks, *i.e*. bigger the parameter *β*_*is*_, bigger is the contribution of internal standard's *s *variability to the normalization correction factor of metabolite peak *i*.

From the equations (3–5) it follows that *Y*_*ij *_can be modeled as normally distributed,

*Y*_*ij *_~ *N*(*μ*_*i *_+ *ρ*_*ij*_, σi2
 MathType@MTEF@5@5@+=feaafiart1ev1aaatCvAUfKttLearuWrP9MDH5MBPbIqV92AaeXatLxBI9gBaebbnrfifHhDYfgasaacH8akY=wiFfYdH8Gipec8Eeeu0xXdbba9frFj0=OqFfea0dXdd9vqai=hGuQ8kuc9pgc9s8qqaq=dirpe0xb9q8qiLsFr0=vr0=vr0dc8meaabaqaciaacaGaaeqabaqabeGadaaakeaaiiGacqWFdpWCdaqhaaWcbaGaemyAaKgabaGaeGOmaidaaaaa@30F0@),     (6)

therefore the log likelihood *L *of observing data **Y **under the assumption of normality is

ℒ=log⁡(∏ijP(Yij|μi,ρij,σi2))==−12∑ij(log⁡(2πσi2)+(Yij−μi−∑sβis(Ωsj−〈Ωs.〉))2σi2) .     (7)
 MathType@MTEF@5@5@+=feaafiart1ev1aaatCvAUfKttLearuWrP9MDH5MBPbIqV92AaeXatLxBI9gBaebbnrfifHhDYfgasaacH8akY=wiFfYdH8Gipec8Eeeu0xXdbba9frFj0=OqFfea0dXdd9vqai=hGuQ8kuc9pgc9s8qqaq=dirpe0xb9q8qiLsFr0=vr0=vr0dc8meaabaqaciaacaGaaeqabaqabeGadaaakeaat0uy0HwzTfgDPnwy1egaryqtHrhAL1wy0L2yHvdaiqaacqWFsectcqGH9aqpcyGGSbaBcqGGVbWBcqGGNbWzdaqadaqaamaarafabaGaemiuaa1aaeWaaeaacqWGzbqwdaWgaaWcbaGaemyAaKMaemOAaOgabeaakiabcYha8HGaciab+X7aTnaaBaaaleaacqWGPbqAaeqaaOGaeiilaWIae4xWdi3aaSbaaSqaaiabdMgaPjabdQgaQbqabaGccqGGSaalcqGFdpWCdaqhaaWcbaGaemyAaKgabaGaeGOmaidaaaGccaGLOaGaayzkaaaaleaacqWGPbqAcqWGQbGAaeqaniabg+GivdaakiaawIcacaGLPaaacqGH9aqpcqGH9aqpcqGHsisldaWcaaqaaiabigdaXaqaaiabikdaYaaadaaeqbqaamaabmaabaGagiiBaWMaei4Ba8Maei4zaC2aaeWaaeaacqaIYaGmcqGFapaCcqGFdpWCdaqhaaWcbaGaemyAaKgabaGaeGOmaidaaaGccaGLOaGaayzkaaGaey4kaSYaaSaaaeaadaqadaqaaiabdMfaznaaBaaaleaacqWGPbqAcqWGQbGAaeqaaOGaeyOeI0Iae4hVd02aaSbaaSqaaiabdMgaPbqabaGccqGHsisldaaeqbqaaiab+j7aInaaBaaaleaacqWGPbqAcqWGZbWCaeqaaOWaaeWaaeaacqqHPoWvdaWgaaWcbaGaem4CamNaemOAaOgabeaakiabgkHiTmaaamaabaGaeuyQdC1aaSbaaSqaaiabdohaZjabc6caUaqabaaakiaawMYicaGLQmcaaiaawIcacaGLPaaaaSqaaiabdohaZbqab0GaeyyeIuoaaOGaayjkaiaawMcaamaaCaaaleqabaGaeGOmaidaaaGcbaGae43Wdm3aa0baaSqaaiabdMgaPbqaaiabikdaYaaaaaaakiaawIcacaGLPaaaaSqaaiabdMgaPjabdQgaQbqab0GaeyyeIuoakiabbccaGiabb6caUiaaxMaacaWLjaWaaeWaaeaacqqG3aWnaiaawIcacaGLPaaaaaa@9CE2@

We note that the simple form of Equation (7) is due to the assumption of independence of the random errors in Equation (4), both across different sample measurements and across different metabolites. While the former assumption is easy to accept, the latter assumption is arguable, because it is well known that co-regulated metabolites are highly correlated [18]. However, in order to keep the number of parameters in the model moderate, we decided to adhere to the latter assumption, being aware of its possible effect on the precision of the parameter estimates [19].

We solve for the values of parameters *μ*_*i*_, *β*_*is*_, and σi2
 MathType@MTEF@5@5@+=feaafiart1ev1aaatCvAUfKttLearuWrP9MDH5MBPbIqV92AaeXatLxBI9gBaebbnrfifHhDYfgasaacH8akY=wiFfYdH8Gipec8Eeeu0xXdbba9frFj0=OqFfea0dXdd9vqai=hGuQ8kuc9pgc9s8qqaq=dirpe0xb9q8qiLsFr0=vr0=vr0dc8meaabaqaciaacaGaaeqabaqabeGadaaakeaaiiGacqWFdpWCdaqhaaWcbaGaemyAaKgabaGaeGOmaidaaaaa@30F0@ that maximize the log likelihood of observing the data:

arg⁡max⁡μi,βis,σi2ℒ
 MathType@MTEF@5@5@+=feaafiart1ev1aaatCvAUfKttLearuWrP9MDH5MBPbIqV92AaeXatLxBI9gBaebbnrfifHhDYfgasaacH8akY=wiFfYdH8Gipec8Eeeu0xXdbba9frFj0=OqFfea0dXdd9vqai=hGuQ8kuc9pgc9s8qqaq=dirpe0xb9q8qiLsFr0=vr0=vr0dc8meaabaqaciaacaGaaeqabaqabeGadaaakeaadaWfqaqaaiGbcggaHjabckhaYjabcEgaNjGbc2gaTjabcggaHjabcIha4bWcbaacciGae8hVd02aaSbaaWqaaiabdMgaPbqabaWccqGGSaalcqWFYoGydaWgaaadbaGaemyAaKMaem4CamhabeaaliabcYcaSiab=n8aZnaaDaaameaacqWGPbqAaeaacqaIYaGmaaaaleqaamrtHrhAL1wy0L2yHvtyaeHbnfgDOvwBHrxAJfwnaGabaOGae4NeHWeaaa@4DD5@

Setting ∂ℒ
 MathType@MTEF@5@5@+=feaafiart1ev1aaatCvAUfKttLearuWrP9MDH5MBPbIqV92AaeXatLxBI9gBamrtHrhAL1wy0L2yHvtyaeHbnfgDOvwBHrxAJfwnaebbnrfifHhDYfgasaacH8akY=wiFfYdH8Gipec8Eeeu0xXdbba9frFj0=OqFfea0dXdd9vqai=hGuQ8kuc9pgc9s8qqaq=dirpe0xb9q8qiLsFr0=vr0=vr0dc8meaabaqaciaacaGaaeqabaWaaeGaeaaakeaaimaacqWFsectaaa@376E@/∂*μ*_*i *_= 0, ∂ℒ
 MathType@MTEF@5@5@+=feaafiart1ev1aaatCvAUfKttLearuWrP9MDH5MBPbIqV92AaeXatLxBI9gBamrtHrhAL1wy0L2yHvtyaeHbnfgDOvwBHrxAJfwnaebbnrfifHhDYfgasaacH8akY=wiFfYdH8Gipec8Eeeu0xXdbba9frFj0=OqFfea0dXdd9vqai=hGuQ8kuc9pgc9s8qqaq=dirpe0xb9q8qiLsFr0=vr0=vr0dc8meaabaqaciaacaGaaeqabaWaaeGaeaaakeaaimaacqWFsectaaa@376E@/∂*β*_*is *_= 0, and ∂ℒ
 MathType@MTEF@5@5@+=feaafiart1ev1aaatCvAUfKttLearuWrP9MDH5MBPbIqV92AaeXatLxBI9gBamrtHrhAL1wy0L2yHvtyaeHbnfgDOvwBHrxAJfwnaebbnrfifHhDYfgasaacH8akY=wiFfYdH8Gipec8Eeeu0xXdbba9frFj0=OqFfea0dXdd9vqai=hGuQ8kuc9pgc9s8qqaq=dirpe0xb9q8qiLsFr0=vr0=vr0dc8meaabaqaciaacaGaaeqabaWaaeGaeaaakeaaimaacqWFsectaaa@376E@/∂σ_i_ = 0 leads to the following equations:

μi=1M(∑jYij−∑j,sβis(Ωsj−〈Ωs.〉)),     (9)
 MathType@MTEF@5@5@+=feaafiart1ev1aaatCvAUfKttLearuWrP9MDH5MBPbIqV92AaeXatLxBI9gBaebbnrfifHhDYfgasaacH8akY=wiFfYdH8Gipec8Eeeu0xXdbba9frFj0=OqFfea0dXdd9vqai=hGuQ8kuc9pgc9s8qqaq=dirpe0xb9q8qiLsFr0=vr0=vr0dc8meaabaqaciaacaGaaeqabaqabeGadaaakeaaiiGacqWF8oqBdaWgaaWcbaGaemyAaKgabeaakiabg2da9maalaaabaGaeGymaedabaGaemyta0eaamaabmaabaWaaabuaeaacqWGzbqwdaWgaaWcbaGaemyAaKMaemOAaOgabeaaaeaacqWGQbGAaeqaniabggHiLdGccqGHsisldaaeqbqaaiab=j7aInaaBaaaleaacqWGPbqAcqWGZbWCaeqaaOWaaeWaaeaacqqHPoWvdaWgaaWcbaGaem4CamNaemOAaOgabeaakiabgkHiTmaaamaabaGaeuyQdC1aaSbaaSqaaiabdohaZjabc6caUaqabaaakiaawMYicaGLQmcaaiaawIcacaGLPaaaaSqaaiabdQgaQjabcYcaSiabdohaZbqab0GaeyyeIuoaaOGaayjkaiaawMcaaiabcYcaSiaaxMaacaWLjaWaaeWaaeaacqaI5aqoaiaawIcacaGLPaaaaaa@5946@

∑j(Yij−μi)(Ωsj−〈Ωs.〉)=∑tβit(Ωtj−〈Ωt.〉)(Ωsj−〈Ωs.〉), and     (10)
 MathType@MTEF@5@5@+=feaafiart1ev1aaatCvAUfKttLearuWrP9MDH5MBPbIqV92AaeXatLxBI9gBaebbnrfifHhDYfgasaacH8akY=wiFfYdH8Gipec8Eeeu0xXdbba9frFj0=OqFfea0dXdd9vqai=hGuQ8kuc9pgc9s8qqaq=dirpe0xb9q8qiLsFr0=vr0=vr0dc8meaabaqaciaacaGaaeqabaqabeGadaaakeaadaaeqbqaamaabmaabaGaemywaK1aaSbaaSqaaiabdMgaPjabdQgaQbqabaGccqGHsisliiGacqWF8oqBdaWgaaWcbaGaemyAaKgabeaaaOGaayjkaiaawMcaaaWcbaGaemOAaOgabeqdcqGHris5aOWaaeWaaeaacqqHPoWvdaWgaaWcbaGaem4CamNaemOAaOgabeaakiabgkHiTmaaamaabaGaeuyQdC1aaSbaaSqaaiabdohaZjabc6caUaqabaaakiaawMYicaGLQmcaaiaawIcacaGLPaaacqGH9aqpdaaeqbqaaiab=j7aInaaBaaaleaacqWGPbqAcqWG0baDaeqaaOWaaeWaaeaacqqHPoWvdaWgaaWcbaGaemiDaqNaemOAaOgabeaakiabgkHiTmaaamaabaGaeuyQdC1aaSbaaSqaaiabdsha0jabc6caUaqabaaakiaawMYicaGLQmcaaiaawIcacaGLPaaadaqadaqaaiabfM6axnaaBaaaleaacqWGZbWCcqWGQbGAaeqaaOGaeyOeI0YaaaWaaeaacqqHPoWvdaWgaaWcbaGaem4CamNaeiOla4cabeaaaOGaayzkJiaawQYiaaGaayjkaiaawMcaaaWcbaGaemiDaqhabeqdcqGHris5aOGaeiilaWIaeeiiaaIaeeyyaeMaeeOBa4MaeeizaqMaaCzcaiaaxMaadaqadaqaaiabbgdaXiabbcdaWaGaayjkaiaawMcaaaaa@746F@

σi2=1M∑j(Yij−μi−∑sβis(Ωsj−〈Ωs.〉))2.     (11)
 MathType@MTEF@5@5@+=feaafiart1ev1aaatCvAUfKttLearuWrP9MDH5MBPbIqV92AaeXatLxBI9gBaebbnrfifHhDYfgasaacH8akY=wiFfYdH8Gipec8Eeeu0xXdbba9frFj0=OqFfea0dXdd9vqai=hGuQ8kuc9pgc9s8qqaq=dirpe0xb9q8qiLsFr0=vr0=vr0dc8meaabaqaciaacaGaaeqabaqabeGadaaakeaaiiGacqWFdpWCdaqhaaWcbaGaemyAaKgabaGaeGOmaidaaOGaeyypa0ZaaSaaaeaacqaIXaqmaeaacqWGnbqtaaWaaabuaeaadaqadaqaaiabdMfaznaaBaaaleaacqWGPbqAcqWGQbGAaeqaaOGaeyOeI0Iae8hVd02aaSbaaSqaaiabdMgaPbqabaGccqGHsisldaaeqbqaaiab=j7aInaaBaaaleaacqWGPbqAcqWGZbWCaeqaaOWaaeWaaeaacqqHPoWvdaWgaaWcbaGaem4CamNaemOAaOgabeaakiabgkHiTmaaamaabaGaeuyQdC1aaSbaaSqaaiabdohaZjabc6caUaqabaaakiaawMYicaGLQmcaaiaawIcacaGLPaaaaSqaaiabdohaZbqab0GaeyyeIuoaaOGaayjkaiaawMcaamaaCaaaleqabaGaeGOmaidaaaqaaiabdQgaQbqab0GaeyyeIuoakiabc6caUiaaxMaacaWLjaWaaeWaaeaacqaIXaqmcqaIXaqmaiaawIcacaGLPaaaaaa@5E45@

Since ∑jΩsj≡∑j〈Ωs.〉
 MathType@MTEF@5@5@+=feaafiart1ev1aaatCvAUfKttLearuWrP9MDH5MBPbIqV92AaeXatLxBI9gBaebbnrfifHhDYfgasaacH8akY=wiFfYdH8Gipec8Eeeu0xXdbba9frFj0=OqFfea0dXdd9vqai=hGuQ8kuc9pgc9s8qqaq=dirpe0xb9q8qiLsFr0=vr0=vr0dc8meaabaqaciaacaGaaeqabaqabeGadaaakeaadaaeqaqaaiabfM6axnaaBaaaleaacqWGZbWCcqWGQbGAaeqaaaqaaiabdQgaQbqab0GaeyyeIuoakiabggMi6oaaqababaWaaaWaaeaacqqHPoWvdaWgaaWcbaGaem4CamNaeiOla4cabeaaaOGaayzkJiaawQYiaaWcbaGaemOAaOgabeqdcqGHris5aaaa@3F61@, the Equation (9) leads to

*μ*_*i *_= 〈Y_*i*._〉.     (12)

The Equation (10) can be written as a matrix product:

Ξis=∑tβitΣts ,     (13)
 MathType@MTEF@5@5@+=feaafiart1ev1aaatCvAUfKttLearuWrP9MDH5MBPbIqV92AaeXatLxBI9gBaebbnrfifHhDYfgasaacH8akY=wiFfYdH8Gipec8Eeeu0xXdbba9frFj0=OqFfea0dXdd9vqai=hGuQ8kuc9pgc9s8qqaq=dirpe0xb9q8qiLsFr0=vr0=vr0dc8meaabaqaciaacaGaaeqabaqabeGadaaakeaacqqHEoawdaWgaaWcbaGaeeyAaKMaee4Camhabeaakiabg2da9maaqafabaacciGae8NSdi2aaSbaaSqaaiabbMgaPjabbsha0bqabaGccqqHJoWudaWgaaWcbaGaemiDaqNaem4CamhabeaaaeaacqqG0baDaeqaniabggHiLdGccqqGGaaicqqGSaalcaWLjaGaaCzcamaabmaabaGaeeymaeJaee4mamdacaGLOaGaayzkaaaaaa@4542@

where

Ξis=∑j(Yij−〈Yi.〉)(Ωsj−〈Ωs.〉)     (14)
 MathType@MTEF@5@5@+=feaafiart1ev1aaatCvAUfKttLearuWrP9MDH5MBPbIqV92AaeXatLxBI9gBaebbnrfifHhDYfgasaacH8akY=wiFfYdH8Gipec8Eeeu0xXdbba9frFj0=OqFfea0dXdd9vqai=hGuQ8kuc9pgc9s8qqaq=dirpe0xb9q8qiLsFr0=vr0=vr0dc8meaabaqaciaacaGaaeqabaqabeGadaaakeaacqqHEoawdaWgaaWcbaGaemyAaKMaem4Camhabeaakiabg2da9maaqafabaWaaeWaaeaacqWGzbqwdaWgaaWcbaGaemyAaKMaemOAaOgabeaakiabgkHiTmaaamaabaGaemywaK1aaSbaaSqaaiabdMgaPjabc6caUaqabaaakiaawMYicaGLQmcaaiaawIcacaGLPaaaaSqaaiabdQgaQbqab0GaeyyeIuoakmaabmaabaGaeuyQdC1aaSbaaSqaaiabdohaZjabdQgaQbqabaGccqGHsisldaaadaqaaiabfM6axnaaBaaaleaacqWGZbWCcqGGUaGlaeqaaaGccaGLPmIaayPkJaaacaGLOaGaayzkaaGaaCzcaiaaxMaadaqadaqaaiabigdaXiabisda0aGaayjkaiaawMcaaaaa@537F@

correlates internal standards and endogenous metabolite peaks, while

Σts=∑j(Ωtj−〈Ωt.〉)(Ωsj−〈Ωs.〉)     (15)
 MathType@MTEF@5@5@+=feaafiart1ev1aaatCvAUfKttLearuWrP9MDH5MBPbIqV92AaeXatLxBI9gBaebbnrfifHhDYfgasaacH8akY=wiFfYdH8Gipec8Eeeu0xXdbba9frFj0=OqFfea0dXdd9vqai=hGuQ8kuc9pgc9s8qqaq=dirpe0xb9q8qiLsFr0=vr0=vr0dc8meaabaqaciaacaGaaeqabaqabeGadaaakeaacqqHJoWudaWgaaWcbaGaemiDaqNaem4Camhabeaakiabg2da9maaqafabaWaaeWaaeaacqqHPoWvdaWgaaWcbaGaemiDaqNaemOAaOgabeaakiabgkHiTmaaamaabaGaeuyQdC1aaSbaaSqaaiabdsha0jabc6caUaqabaaakiaawMYicaGLQmcaaiaawIcacaGLPaaaaSqaaiabdQgaQbqab0GaeyyeIuoakmaabmaabaGaeuyQdC1aaSbaaSqaaiabdohaZjabdQgaQbqabaGccqGHsisldaaadaqaaiabfM6axnaaBaaaleaacqWGZbWCcqGGUaGlaeqaaaGccaGLPmIaayPkJaaacaGLOaGaayzkaaGaaCzcaiaaxMaadaqadaqaaiabigdaXiabiwda1aGaayjkaiaawMcaaaaa@5469@

is a covariance matrix for the internal standards. Multiplying each side of Equation (13) by the inverse of matrix **Σ**, the estimates for the parameters **β **can be written as a product of two matrices:

β^=Ξ^×Σ−1,     (16)
 MathType@MTEF@5@5@+=feaafiart1ev1aaatCvAUfKttLearuWrP9MDH5MBPbIqV92AaeXatLxBI9gBaebbnrfifHhDYfgasaacH8akY=wiFfYdH8Gipec8Eeeu0xXdbba9frFj0=OqFfea0dXdd9vqai=hGuQ8kuc9pgc9s8qqaq=dirpe0xb9q8qiLsFr0=vr0=vr0dc8meaabaqaciaacaGaaeqabaqabeGadaaakeaaiiqacuWFYoGygaqcaiabg2da9iqb=55ayzaajaGaey41aqRae83Odm1aaWbaaSqabeaaiiaacqGFsislcqGFXaqmaaGccqqGSaalcaWLjaGaaCzcamaabmaabaGaeeymaeJaeeOnaydacaGLOaGaayzkaaaaaa@3C20@

where the hat notation means that the matrices are evaluated using the actually observed data Y. Based on the multiplicative error model from Equation(1), the normalized intensities for each peak are then calculated as

X˜ij=Xij×exp⁡(−∑s=1Sβ^is(Ωsj−〈Ωs.〉)).     (17)
 MathType@MTEF@5@5@+=feaafiart1ev1aaatCvAUfKttLearuWrP9MDH5MBPbIqV92AaeXatLxBI9gBaebbnrfifHhDYfgasaacH8akY=wiFfYdH8Gipec8Eeeu0xXdbba9frFj0=OqFfea0dXdd9vqai=hGuQ8kuc9pgc9s8qqaq=dirpe0xb9q8qiLsFr0=vr0=vr0dc8meaabaqaciaacaGaaeqabaqabeGadaaakeaacuWGybawgaacamaaBaaaleaacqWGPbqAcqWGQbGAaeqaaOGaeyypa0JaemiwaG1aaSbaaSqaaiabdMgaPjabdQgaQbqabaGccqGHxdaTcyGGLbqzcqGG4baEcqGGWbaCdaqadaqaaiabgkHiTmaaqahabaacciGaf8NSdiMbaKaadaWgaaWcbaGaemyAaKMaem4CamhabeaakmaabmaabaGaeuyQdC1aaSbaaSqaaiabdohaZjabdQgaQbqabaGccqGHsisldaaadaqaaiabfM6axnaaBaaaleaacqWGZbWCcqGGUaGlaeqaaaGccaGLPmIaayPkJaaacaGLOaGaayzkaaaaleaacqWGZbWCcqGH9aqpcqaIXaqmaeaacqWGtbWua0GaeyyeIuoaaOGaayjkaiaawMcaaiabb6caUiaaxMaacaWLjaWaaeWaaeaacqqGXaqmcqqG3aWnaiaawIcacaGLPaaaaaa@5CE3@

Once the model has been trained, i.e., the parameter **β **has been estimated, it can be applied to a new data of samples from a similar biological experiment with arbitrary true metabolite levels, that is, in Equation 1 the true level, *m*_*ij*_, can be sample dependent.

### Normalization in case of a single internal standard

In order to present an intuitive example of how the NOMIS method works, we demonstrate it in a hypothetical case of a single internal standard. In this case the intensity matrix of internal standard peaks **Z **is a 1 × *M *vector (*Z*_1*j*_)_*j *= 1..*M *_and the Equations

(16) and (17) straightforwardly lead to:

X˜ij=Xij×((∏k=1MZ1k)1MZ1j)ri1σ12;     (18)
 MathType@MTEF@5@5@+=feaafiart1ev1aaatCvAUfKttLearuWrP9MDH5MBPbIqV92AaeXatLxBI9gBaebbnrfifHhDYfgasaacH8akY=wiFfYdH8Gipec8Eeeu0xXdbba9frFj0=OqFfea0dXdd9vqai=hGuQ8kuc9pgc9s8qqaq=dirpe0xb9q8qiLsFr0=vr0=vr0dc8meaabaqaciaacaGaaeqabaqabeGadaaakeaacuWGybawgaacamaaBaaaleaacqWGPbqAcqWGQbGAaeqaaOGaeyypa0JaemiwaG1aaSbaaSqaaiabdMgaPjabdQgaQbqabaGccqGHxdaTdaqadaqaamaalaaabaWaaeWaaeaadaqeWbqaaiabdQfaAnaaBaaaleaacqaIXaqmcqWGRbWAaeqaaaqaaiabdUgaRjabg2da9iabigdaXaqaaiabd2eanbqdcqGHpis1aaGccaGLOaGaayzkaaWaaWbaaSqabeaadaWcaaqaaiabigdaXaqaaiabd2eanbaaaaaakeaacqWGAbGwdaWgaaWcbaGaeGymaeJaemOAaOgabeaaaaaakiaawIcacaGLPaaadaahaaWcbeqaamaalaaabaGaemOCai3aaSbaaWqaaiabdMgaPjabigdaXaqabaaaleaaiiGacqWFdpWCdaqhaaadbaGaeGymaedabaGaeGOmaidaaaaaaaGccqGG7aWocaWLjaGaaCzcamaabmaabaGaeGymaeJaeGioaGdacaGLOaGaayzkaaaaaa@598C@

where

ri1=∑j=1M(log⁡Xij−〈log⁡Xi.〉)(log⁡Z1j−〈log⁡Z1.〉), and     (19)
 MathType@MTEF@5@5@+=feaafiart1ev1aaatCvAUfKttLearuWrP9MDH5MBPbIqV92AaeXatLxBI9gBaebbnrfifHhDYfgasaacH8akY=wiFfYdH8Gipec8Eeeu0xXdbba9frFj0=OqFfea0dXdd9vqai=hGuQ8kuc9pgc9s8qqaq=dirpe0xb9q8qiLsFr0=vr0=vr0dc8meaabaqaciaacaGaaeqabaqabeGadaaakeaacqWGYbGCdaWgaaWcbaGaemyAaKMaeGymaedabeaakiabg2da9maaqahabaWaaeWaaeaacyGGSbaBcqGGVbWBcqGGNbWzcqWGybawdaWgaaWcbaGaemyAaKMaemOAaOgabeaakiabgkHiTmaaamaabaGagiiBaWMaei4Ba8Maei4zaCMaemiwaG1aaSbaaSqaaiabdMgaPjabc6caUaqabaaakiaawMYicaGLQmcaaiaawIcacaGLPaaadaqadaqaaiGbcYgaSjabc+gaVjabcEgaNjabdQfaAnaaBaaaleaacqaIXaqmcqWGQbGAaeqaaOGaeyOeI0YaaaWaaeaacyGGSbaBcqGGVbWBcqGGNbWzcqWGAbGwdaWgaaWcbaGaeGymaeJaeiOla4cabeaaaOGaayzkJiaawQYiaaGaayjkaiaawMcaaaWcbaGaemOAaOMaeyypa0JaeGymaedabaGaemyta0eaniabggHiLdGccqGGSaalcqqGGaaicqqGHbqycqqGUbGBcqqGKbazcaWLjaGaaCzcamaabmaabaGaeeymaeJaeeyoaKdacaGLOaGaayzkaaaaaa@6A93@

σ12=∑j=1M(log⁡Z1j−〈log⁡Z1.〉)2.     (20)
 MathType@MTEF@5@5@+=feaafiart1ev1aaatCvAUfKttLearuWrP9MDH5MBPbIqV92AaeXatLxBI9gBaebbnrfifHhDYfgasaacH8akY=wiFfYdH8Gipec8Eeeu0xXdbba9frFj0=OqFfea0dXdd9vqai=hGuQ8kuc9pgc9s8qqaq=dirpe0xb9q8qiLsFr0=vr0=vr0dc8meaabaqaciaacaGaaeqabaqabeGadaaakeaaiiGacqWFdpWCdaqhaaWcbaGaeGymaedabaGaeGOmaidaaOGaeyypa0ZaaabCaeaadaqadaqaaiGbcYgaSjabc+gaVjabcEgaNjabdQfaAnaaBaaaleaacqaIXaqmcqWGQbGAaeqaaOGaeyOeI0YaaaWaaeaacyGGSbaBcqGGVbWBcqGGNbWzcqWGAbGwdaWgaaWcbaGaeGymaeJaeiOla4cabeaaaOGaayzkJiaawQYiaaGaayjkaiaawMcaamaaCaaaleqabaGaeGOmaidaaaqaaiabdQgaQjabg2da9iabigdaXaqaaiabd2eanbqdcqGHris5aOGaeiOla4IaaCzcaiaaxMaadaqadaqaaiabikdaYiabicdaWaGaayjkaiaawMcaaaaa@5285@

We write the internal standard levels as

Log *Z*_1*j *_= *C *+ *ω*_*j*_,     (21)

with *C *= 〈Ω_1._〉 being the mean and *ω*_*j *_deviation of sample j from the mean:

*ω*_*j *_= Ω_1*j *_- 〈Ω_1._〉. We model the endogenous metabolite peaks as:

log *X*_*ij *_= *T*_*i *_+ *β*_*i*_*ω*_*j *_+ *ε*_*ij*_,     (22)

where *T*_*i *_is the true log intensity of metabolite *i*'s peak, *β*_i _is a parameter that describes by how many units the log intensity of peak *i *changes when the log intensity of the standard increases by one unit, and *ε*_ij _is a random error drawn from a normal distribution with zero mean. The coefficients *r*_*i*1 _and σ12
 MathType@MTEF@5@5@+=feaafiart1ev1aaatCvAUfKttLearuWrP9MDH5MBPbIqV92AaeXatLxBI9gBaebbnrfifHhDYfgasaacH8akY=wiFfYdH8Gipec8Eeeu0xXdbba9frFj0=OqFfea0dXdd9vqai=hGuQ8kuc9pgc9s8qqaq=dirpe0xb9q8qiLsFr0=vr0=vr0dc8meaabaqaciaacaGaaeqabaqabeGadaaakeaaiiGacqWFdpWCdaqhaaWcbaGaeGymaedabaGaeGOmaidaaaaa@3085@ from Equations (19) and (20) can then be written as

ri1=βiσi2+∑j=1Mεijωjand     (23)
 MathType@MTEF@5@5@+=feaafiart1ev1aaatCvAUfKttLearuWrP9MDH5MBPbIqV92AaeXatLxBI9gBaebbnrfifHhDYfgasaacH8akY=wiFfYdH8Gipec8Eeeu0xXdbba9frFj0=OqFfea0dXdd9vqai=hGuQ8kuc9pgc9s8qqaq=dirpe0xb9q8qiLsFr0=vr0=vr0dc8meaabaqaciaacaGaaeqabaqabeGadaaakeaafaqabeqacaaabaGaemOCai3aaSbaaSqaaiabdMgaPjabigdaXaqabaGccqGH9aqpiiGacqWFYoGydaWgaaWcbaGaemyAaKgabeaakiab=n8aZnaaDaaaleaacqWGPbqAaeaacqaIYaGmaaGccqGHRaWkdaaeWbqaaiab=v7aLnaaBaaaleaacqWGPbqAcqWGQbGAaeqaaOGae8xYdC3aaSbaaSqaaiabdQgaQbqabaaabaGaemOAaOMaeyypa0JaeGymaedabaGaemyta0eaniabggHiLdaakeaacqqGHbqycqqGUbGBcqqGKbazaaGaaCzcaiaaxMaadaqadaqaaiabikdaYiabiodaZaGaayjkaiaawMcaaaaa@5150@

σ12=∑j=1Mωj2.     (24)
 MathType@MTEF@5@5@+=feaafiart1ev1aaatCvAUfKttLearuWrP9MDH5MBPbIqV92AaeXatLxBI9gBaebbnrfifHhDYfgasaacH8akY=wiFfYdH8Gipec8Eeeu0xXdbba9frFj0=OqFfea0dXdd9vqai=hGuQ8kuc9pgc9s8qqaq=dirpe0xb9q8qiLsFr0=vr0=vr0dc8meaabaqaciaacaGaaeqabaqabeGadaaakeaaiiGacqWFdpWCdaqhaaWcbaGaeGymaedabaGaeGOmaidaaOGaeyypa0ZaaabCaeaacqWFjpWDdaqhaaWcbaGaemOAaOgabaGaeGOmaidaaaqaaiabdQgaQjabg2da9iabigdaXaqaaiabd2eanbqdcqGHris5aOGaeiOla4IaaCzcaiaaxMaadaqadaqaaiabikdaYiabisda0aGaayjkaiaawMcaaaaa@4229@

If we ignore the effect of the residual term in the *r*_*i*1_/σ12
 MathType@MTEF@5@5@+=feaafiart1ev1aaatCvAUfKttLearuWrP9MDH5MBPbIqV92AaeXatLxBI9gBaebbnrfifHhDYfgasaacH8akY=wiFfYdH8Gipec8Eeeu0xXdbba9frFj0=OqFfea0dXdd9vqai=hGuQ8kuc9pgc9s8qqaq=dirpe0xb9q8qiLsFr0=vr0=vr0dc8meaabaqaciaacaGaaeqabaqabeGadaaakeaaiiGacqWFdpWCdaqhaaWcbaGaeGymaedabaGaeGOmaidaaaaa@3085@ ratio:

ri1σ12=βi+ ∑j=1Mεijωj∑j=1Mωj2,     (25)
 MathType@MTEF@5@5@+=feaafiart1ev1aaatCvAUfKttLearuWrP9MDH5MBPbIqV92AaeXatLxBI9gBaebbnrfifHhDYfgasaacH8akY=wiFfYdH8Gipec8Eeeu0xXdbba9frFj0=OqFfea0dXdd9vqai=hGuQ8kuc9pgc9s8qqaq=dirpe0xb9q8qiLsFr0=vr0=vr0dc8meaabaqaciaacaGaaeqabaqabeGadaaakeaadaWcaaqaaiabdkhaYnaaBaaaleaacqWGPbqAcqaIXaqmaeqaaaGcbaacciGae83Wdm3aa0baaSqaaiabigdaXaqaaiabikdaYaaaaaGccqGH9aqpcqWFYoGydaWgaaWcbaGaemyAaKgabeaakiabgUcaRiabbccaGmaalaaabaWaaabCaeaacqWF1oqzdaWgaaWcbaGaemyAaKMaemOAaOgabeaakiab=L8a3naaBaaaleaacqWGQbGAaeqaaaqaaiabdQgaQjabg2da9iabigdaXaqaaiabd2eanbqdcqGHris5aaGcbaWaaabCaeaacqWFjpWDdaqhaaWcbaGaemOAaOgabaGaeGOmaidaaaqaaiabdQgaQjabg2da9iabigdaXaqaaiabd2eanbqdcqGHris5aaaakiabcYcaSiaaxMaacaWLjaWaaeWaaeaacqaIYaGmcqaI1aqnaiaawIcacaGLPaaaaaa@59A3@

then the Equation (18) reduces to

X˜ij≈Xij×(MZ1j)βi,     (26)
 MathType@MTEF@5@5@+=feaafiart1ev1aaatCvAUfKttLearuWrP9MDH5MBPbIqV92AaeXatLxBI9gBaebbnrfifHhDYfgasaacH8akY=wiFfYdH8Gipec8Eeeu0xXdbba9frFj0=OqFfea0dXdd9vqai=hGuQ8kuc9pgc9s8qqaq=dirpe0xb9q8qiLsFr0=vr0=vr0dc8meaabaqaciaacaGaaeqabaqabeGadaaakeaacuWGybawgaacamaaBaaaleaacqWGPbqAcqWGQbGAaeqaaOGaeyisISRaemiwaG1aaSbaaSqaaiabdMgaPjabdQgaQbqabaGccqGHxdaTdaqadaqaamaalaaabaGaemyta0eabaGaemOwaO1aaSbaaSqaaiabigdaXiabdQgaQbqabaaaaaGccaGLOaGaayzkaaWaaWbaaSqabeaaiiGacqWFYoGydaWgaaadbaGaemyAaKgabeaaaaGccqGGSaalcaWLjaGaaCzcamaabmaabaGaeGOmaiJaeGOnaydacaGLOaGaayzkaaaaaa@484D@

Where *M *= exp(*C*). From Equation (16) we see that *β*_*i *_= *c*_*i*_/*c*_11_, with *c*_*i*1 _and *c*_11 _being estimators for the covariance between metabolite i and the standard and the variance of the standard respectively. The interpretation of the result is now straightforward. For example, if *β*_*i *_= 1, *i.e*. the covariance of metabolite *i *with the standard is of the order of the variance of the standard, then the Equation (26) describes a simple correction by the internal standard

X˜ij=MXijZ1j .     (27)
 MathType@MTEF@5@5@+=feaafiart1ev1aaatCvAUfKttLearuWrP9MDH5MBPbIqV92AaeXatLxBI9gBaebbnrfifHhDYfgasaacH8akY=wiFfYdH8Gipec8Eeeu0xXdbba9frFj0=OqFfea0dXdd9vqai=hGuQ8kuc9pgc9s8qqaq=dirpe0xb9q8qiLsFr0=vr0=vr0dc8meaabaqaciaacaGaaeqabaqabeGadaaakeaacuWGybawgaacamaaBaaaleaacqWGPbqAcqWGQbGAaeqaaOGaeyypa0Jaemyta00aaSaaaeaacqWGybawdaWgaaWcbaGaemyAaKMaemOAaOgabeaaaOqaaiabdQfaAnaaBaaaleaacqaIXaqmcqWGQbGAaeqaaaaakiabbccaGiabb6caUiaaxMaacaWLjaWaaeWaaeaacqqGYaGmcqqG3aWnaiaawIcacaGLPaaaaaa@4159@

Such correction is commonly applied to specific metabolites when their corresponding isotope labeled standards are available. In contrast, if a specific metabolite is uncorrelated to the internal standard, *β*_*i *_= 0, and the normalization factor is 1, leading to X˜
 MathType@MTEF@5@5@+=feaafiart1ev1aaatCvAUfKttLearuWrP9MDH5MBPbIqV92AaeXatLxBI9gBaebbnrfifHhDYfgasaacH8akY=wiFfYdH8Gipec8Eeeu0xXdbba9frFj0=OqFfea0dXdd9vqai=hGuQ8kuc9pgc9s8qqaq=dirpe0xb9q8qiLsFr0=vr0=vr0dc8meaabaqaciaacaGaaeqabaqabeGadaaakeaacuWGybawgaacaaaa@2DF4@_*ij *_= *X*_*ij*_. Thus, if the linear association between a metabolite and the standard is weak, the NOMIS method reduces the extent of normalization.

In the following section we demonstrate the NOMIS method using the multiple internal standard applications in real biological samples.

### Method performance and comparison to other methods: mouse liver lipidomics dataset

In order to evaluate the performance of the NOMIS method, we performed UPLC/MS analysis of mouse liver using lipidomics platform as described in Methods. We run 16 replicates of the same biological sample in repeatability conditions, corresponding to 3 different extracts of 10, 3, and 3 sample injections each, respectively. Total 5 internal standard compounds of distinct chemical and functional characteristics were injected prior to extraction. Total 1470 monoisotopic peaks were detected using MZmine [20] software version 0.60. The processed dataset with partial identification is available as Additional file 1 and the internal standards are listed in Table 1.

**Table 1:** Lipid internal standards The list of internal standards utilized in the demonstrations of the paper, their abbreviations, common names, amount in the sample, retention time in the UPLC/MS method described in the Methods, mean intensity as peak height, and coefficient of variance based on the 16-run liver repeatability study.

**Abbreviation**	**Name**	**Amount (*μ*g/sample)**	**Retention time (s)**	**Mean Intensity**	**CV**
LPC	GPCho(17:0/0:0)	6.408	210	5574	0.118
Cer	Cer(d18:1/17:0)	1.832	381	1044	0.197
PC	GPCho(17:0/17:0)	0.198	388	521	0.111
PE	GPEth(17:0/17:0)	1.790	392	316	0.134
TAG	TG(17:0/17:0/17:0)	2.072	543	202	0.335

The performance of the NOMIS method is compared to two other methods. The first is a commonly utilized normalization by *l*_2 _vector norm (abbreviated as *L2N*) [10]:

X˜ij=Xij×〈∑i=1...NXi.2〉∑i=1...NXij2 ;     (28)
 MathType@MTEF@5@5@+=feaafiart1ev1aaatCvAUfKttLearuWrP9MDH5MBPbIqV92AaeXatLxBI9gBaebbnrfifHhDYfgasaacH8akY=wiFfYdH8Gipec8Eeeu0xXdbba9frFj0=OqFfea0dXdd9vqai=hGuQ8kuc9pgc9s8qqaq=dirpe0xb9q8qiLsFr0=vr0=vr0dc8meaabaqaciaacaGaaeqabaqabeGadaaakeaacuWGybawgaacamaaBaaaleaacqWGPbqAcqWGQbGAaeqaaOGaeyypa0JaemiwaG1aaSbaaSqaaiabdMgaPjabdQgaQbqabaGccqGHxdaTdaGcaaqaamaalaaabaWaaaWaaeaadaaeqbqaaiabdIfaynaaDaaaleaacqWGPbqAcqGGUaGlaeaacqaIYaGmaaaabaGaemyAaKMaeyypa0JaeGymaeJaeiOla4IaeiOla4IaeiOla4IaemOta4eabeqdcqGHris5aaGccaGLPmIaayPkJaaabaWaaabuaeaacqWGybawdaqhaaWcbaGaemyAaKMaemOAaOgabaGaeGOmaidaaaqaaiabdMgaPjabg2da9iabigdaXiabc6caUiabc6caUiabc6caUiabd6eaobqab0GaeyyeIuoaaaaaleqaaOGaeeiiaaIaee4oaSJaaCzcaiaaxMaadaqadaqaaiabbkdaYiabbIda4aGaayjkaiaawMcaaaaa@5CC3@

where the average 〈 〉 is taken over the samples *j *= 1...*M*. The second method is essentially the same as in [14], based on the application of three internal standard compounds (*3STD*) with the choices of retention time ranges reflecting the analytical method used: LPC standard is applied for peaks with *rt *< 300s, PC standard for 300s <*rt *< 410s, and TAG standard for *rt *> 410s.

We utilize coefficient of variation (CV) as the main performance measure for normalization methods. The CV is defined as the ratio of the standard deviation and the mean:

CVi=(〈Yi.2〉−〈Yi.〉2)〈Yi.〉 .     (26)
 MathType@MTEF@5@5@+=feaafiart1ev1aaatCvAUfKttLearuWrP9MDH5MBPbIqV92AaeXatLxBI9gBaebbnrfifHhDYfgasaacH8akY=wiFfYdH8Gipec8Eeeu0xXdbba9frFj0=OqFfea0dXdd9vqai=hGuQ8kuc9pgc9s8qqaq=dirpe0xb9q8qiLsFr0=vr0=vr0dc8meaabaqaciaacaGaaeqabaqabeGadaaakeaacqWGdbWqcqWGwbGvdaWgaaWcbaGaemyAaKgabeaakiabg2da9maalaaabaWaaOaaaeaadaqadaqaamaaamaabaGaemywaK1aa0baaSqaaiabdMgaPjabc6caUaqaaiabikdaYaaaaOGaayzkJiaawQYiaiabgkHiTmaaamaabaGaemywaK1aaSbaaSqaaiabdMgaPjabc6caUaqabaaakiaawMYicaGLQmcadaahaaWcbeqaaiabikdaYaaaaOGaayjkaiaawMcaaaWcbeaaaOqaamaaamaabaGaemywaK1aaSbaaSqaaiabdMgaPjabc6caUaqabaaakiaawMYicaGLQmcaaaGaeeiiaaIaeeOla4IaaCzcaiaaxMaadaqadaqaaiabbkdaYiabbAda2aGaayjkaiaawMcaaaaa@4D23@

As the overall measure of variability we apply median CV:

*MCV *≡ median {*CV*_*i*_}_*i *=1...*N *_    (30)

**Figure 3:**
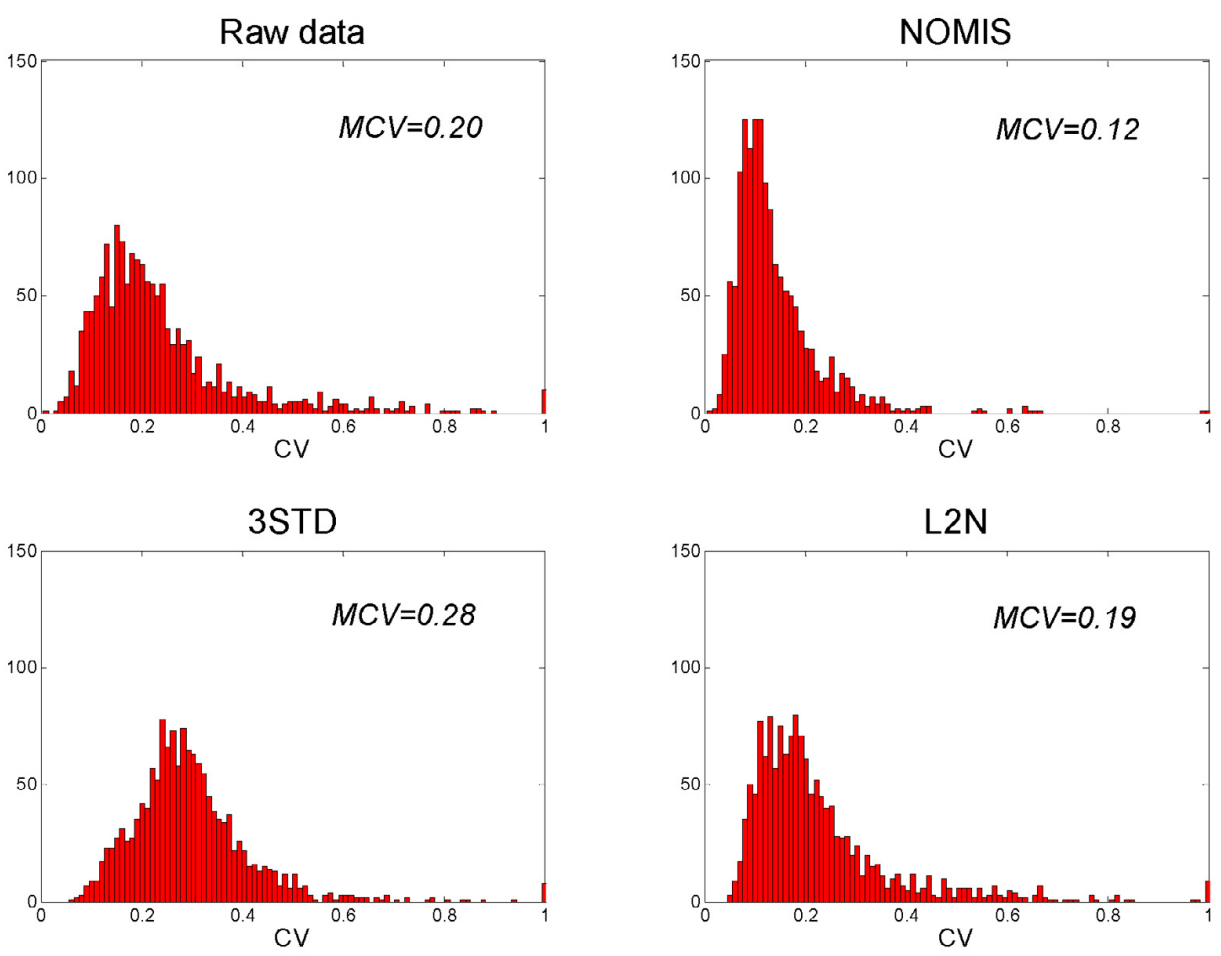
**Coefficient of variance distributions for different normalization methods**. Data shown is based on mouse liver run of 16 samples under repeability conditions (3 extractions from the same biological sample, each with repeated runs of 10, 3, and 3 injections, respectively). Total 1470 monoisotopic peaks were included in the analysis. The NOMIS method has a notably narrower CV distribution than raw data and other normalization methods, with lower median CV (MCV).

**Figure 4:**
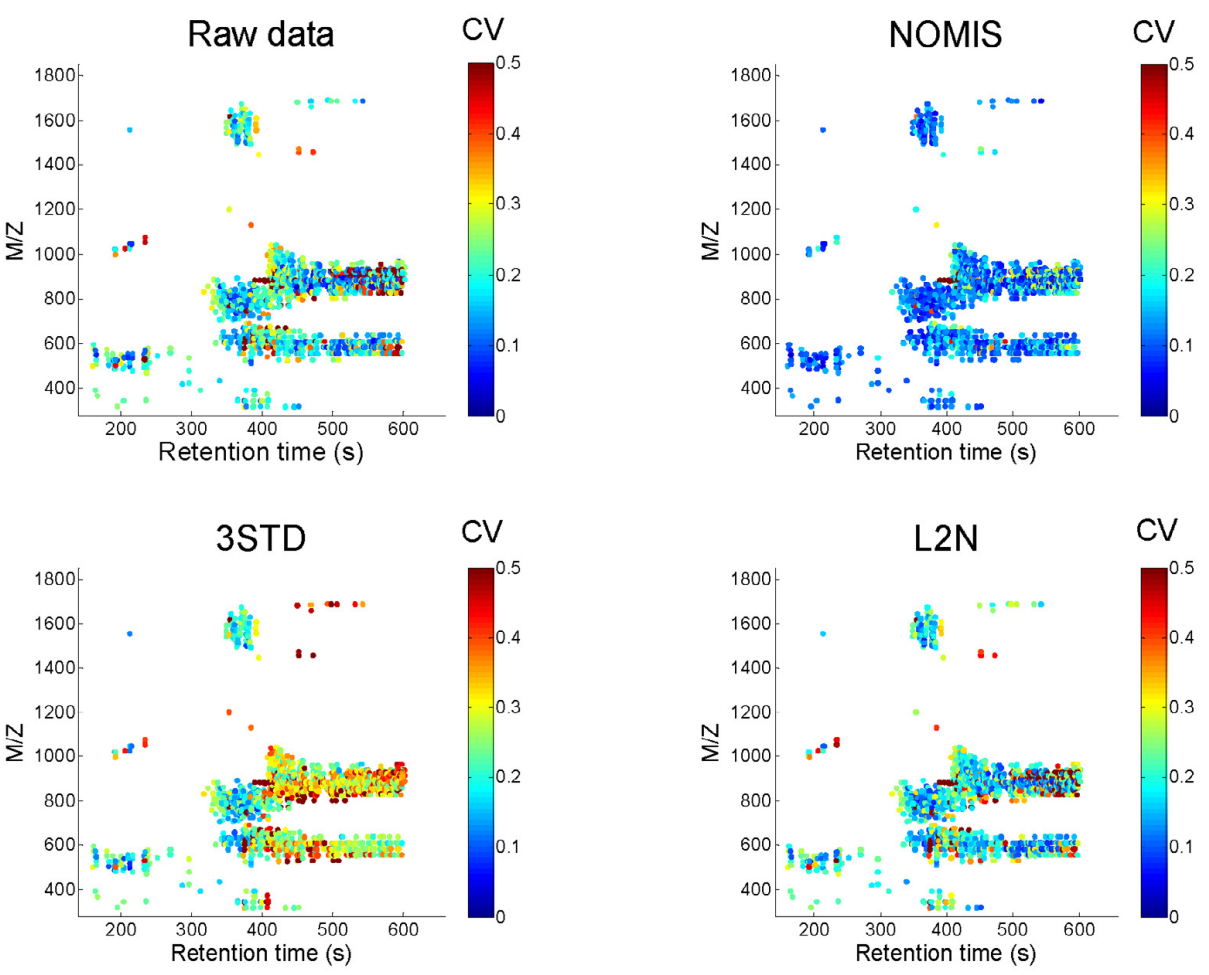
**Coefficients of variance for individual peaks in liver repeatability study**. Each peak detected is shown in the two dimensional plot of m/z *vs*. retention time plot, with the color corresponding to the coefficient of variance. The NOMIS method performs notably better in its ability to reduce the variability across the full spectrum. The 3STD method performs particularly poorly for higher retention times, where the normalization is based on triacylglycerol standard found to be highly variable (Table 1).

Distributions of CV for different normalization methods for a liver lipidomics dataset are shown in Figure 3. The distribution after application of NOMIS method is notably narrower, and the MCV is lower than in raw data or in other normalization methods. Replacement of PC standard with PE in 3STD method did not alter the MCV, with MCV = 0.282 for PE included, compared to MCV = 0.280 with PC. The CVs at individual peak level are shown in Figure 4 in a two-dimensional plot of m/z *vs*. the retention time. One can clearly observe the variability drop across the complete spectrum range for the NOMIS method. In contrast, no specific improvement is observed for the L2N method in any section of the spectrum. The 3STD method performs particularly poorly in the region normalized by the TAG standard, while in other two regions the improvement appears only slight. The TAG standard was found highly variable (Table 1), which we believe is a result of the suppression effects due to high number of different triacylglycerol species in that part of the spectrum. We observed the same problem using the stable isotope labeled external standard TG(16:0/16:0/16:0)13C3 (not shown). Therefore, increased variability of the triacylglycerol standard is not due to variability in sample extraction.

### Heteroscedasticity

Calculation of the **β **matrix using the log transformation is of potential concern because such transformation, while efficient in correcting for heteroscedasticity, may also amplify the high variability of low abundance metabolites [8,21]. The log transformation is also unable to deal with value zero.

**Figure 5:**
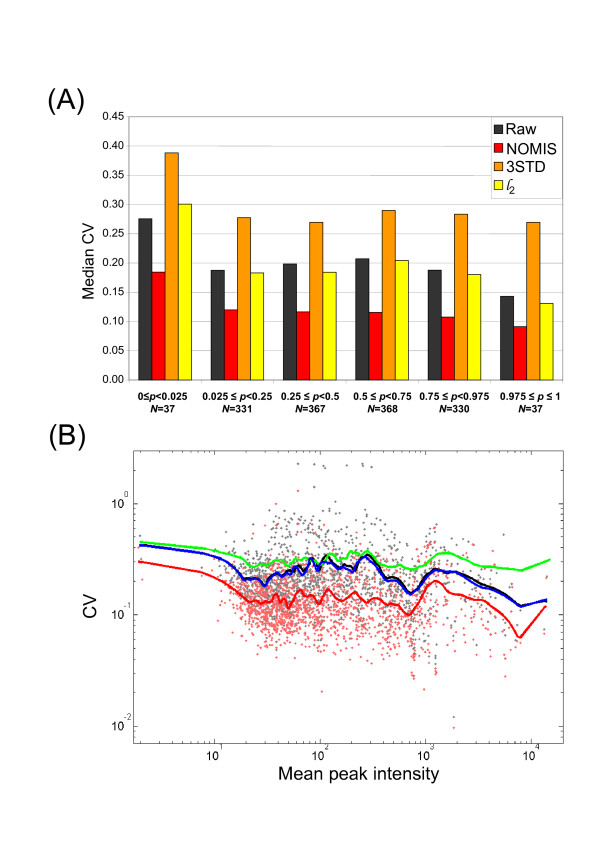
**Effect of normalization methods on heteroscedasticity in the data**. (A) The sorted mean intensity values for 1470 peaks are divided into six bins defined by the quantiles for the following cumulative probabilities: *P*= [0.025, 0.25, 0.5, 0.75, 0.975]. The first bin therefore contains the low abundance peaks on the left tail of the intensity distribution, with cumulative probability *p *< 0.025. The median coefficients of variance were calculated within each bin. (B) Scatter plot of unnormalized and NOMIS normalized peaks. The solid curves, shown for all four methods compared, are drawn as guides using robust locally weighted regression and smoothing scatter plots method (LOWESS) [22] with the 100 variable window size smoothing kernel.

In order to study the effect of different normalization methods on heteroscedasticity in data, we divided the sorted mean intensity values for 1470 peaks into six bins defined by the quantiles for the following cumulative probabilities: *P *= [0.025, 0.25, 0.5, 0.75, 0.975]. The first bin therefore contains the low abundance peaks on the left tail of the intensity distribution, with cumulative probability *p *< 0.025, the second contains peaks with cumulative probability 0.025 = *p *< 0.25, etc. The median coefficients of variance were calculated within each bin and compared across different normalization methods (Figure 5A). The CV for the low abundance peaks (*p *< 0.025) is expectedly highest as compared to other peaks for the unnormalized data as well as for the three normalization methods. The NOMIS method has the lowest MCV within each bin, providing evidence that the use of log transformation in calculation of the **β **matrix does not lead to worse performance for low abundance metabolites relative to the other normalization methods. This can be also seen more directly in the scatter plot of CV and mean values for the unnormalized and NOMIS normalized data (Figure 5B).

We deal with the problem of zeros in log transformation by utilizing the post-alignment peak picking algorithm from the MZmine software [20]. In case of datasets utilized in this paper no exact zeros were found among the 1470 peaks following such processing. Application of the NOMIS method to selection of the internal standard mixture. The systematic study of the results obtained by the NOMIS method can also be utilized for selection of standard compound mixture used in the analytical method. This may be useful in practical analytical work as more standards do not necessarily guarantee better quality of normalization. It is also important to gain understanding of how each individual standard affects the variability of individual molecular species across the full spectra.

**Figure 6:**
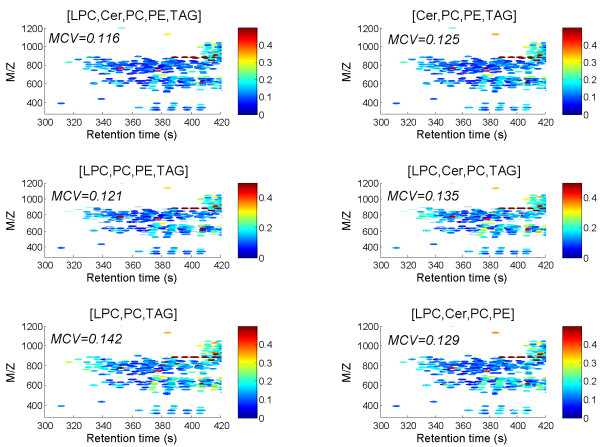
**The NOMIS method as a tool to select the best set of internal standards used for normalization**. The coefficients of variation for different combinations of internal standards used in the NOMIS method as applied to the liver dataset (1470 peaks). Only sub-region of m/z and retention times are shown, corresponding mainly to phospholipids, sphingolipids, and diacylglycerols. The internal standard combinations are listed in the plot titles.

To illustrate such an application of NOMIS, we normalized the liver dataset described above using different combinations of the internal standards. Coefficients of variance within a selected region of m/z and retention time values, corresponding mainly to diacyl-phospholipids, diacylglycerols and sphingolipids, are shown in Figure 6 for six such combinations. From the results one can conclude that the removal of PE standard has the largest negative effect on normalization performance, while the Cer standard has least effect. However, the application of all 5 standards still gives best results. The application of a combination of three standards (LPC, PC, and TAG) performs worse than any of the other combinations shown, with median CV of 0.142. Interestingly, one spectral region where this standard combination performs poorly is for retention times of 3203–340s, where one can find mainly the long fatty acid chain phosphatidylcholines containing high total number of double bonds (see Additional file 1). Therefore, our results suggest the variability (due to systematic error) of the saturated medium fatty acid chain length PC used as a standard is different as for the unsaturated long chain PCs, and therefore is not a good standard for these components. Replacing PC with PE in a three-standard combination (LPC, PE, and TAG) leads to better performance of the NOMIS method, with median CV of 0.127 (figure not shown).

While the variability of the TAG standard is high (CV = 0.335), its inclusion with the other standards still improved the MCV from 0.129 to 0.116. The TAG standard in combination with other standards can therefore model the variability of some metabolites better than the four other standards alone. For example, the correlation of the triacylglycerol levels with the TAG standard is higher than with other standards. The median Pearson correlation coefficient between the internal standard levels and each of the 184 identified TAG species is 0.25, compared to -0.30, 0.17, -0.18, and -0.08 for LPC, Cer, PC, and PE standards, respectively.

The results from analysis of different internal standard combinations above suggest the NOMIS method can be a valuable tool in analytical development. Different biological matrices and different analytical platforms may require different combinations of standards for optimal normalization and systematic evaluation of different standards as illustrated above may provide the necessary clues.

### Investigation of the results in context of the identified molecular species

**Figure 7:**
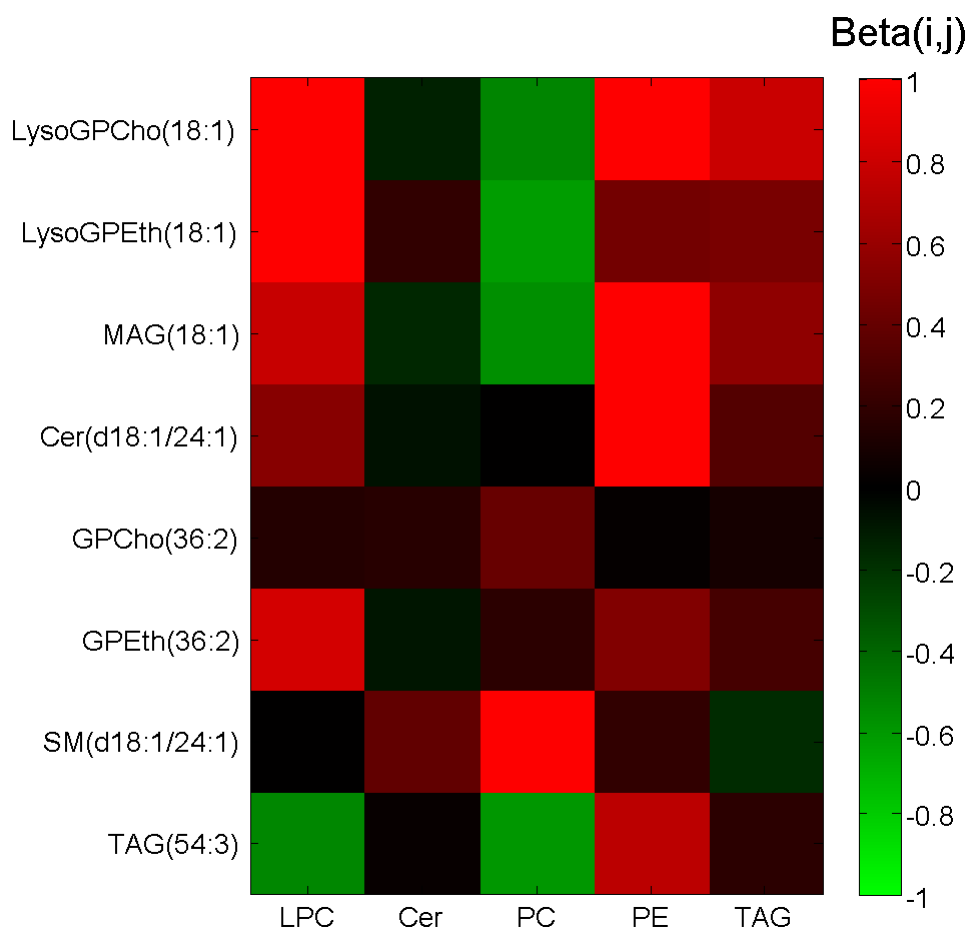
**The *β *matrix values for selected liver lipid components**. The *β *matrix values are shown for 8 illustrative lipid molecular species of different functional class and for all internal standards used (abbreviated as shown in Table 1). The LPC has expectedly high influence on monoacyl lipids. As one would have expected based on chemical structure, the sphingomyelin, which does not have its own class-specific internal standard, is influenced most by ceramide (Cer) and phosphatidylcholine (PC) standards

In order to gain insight into the nature of the NOMIS method in the context of compounds studied, we identified several lipid molecular species in the dataset, 360 in total. As expected, we found that the composition of the **β **matrix correlates well with the composition of lipid functional groups. The elements of **β **for each of the standards are shown in Figure 7 for some of the most abundant representative molecular species from different lipid classes. The normalization factor, dependent both on the **β **matrix and the internal standard concentration, is dominated by the LPC standard for mono-acyl lipid species such as lysophosphatidylcholines, lysophosphatidyletahnolamines, and monoacylglycerols. The sphingomyelin species is affected mostly by a combination of the ceramide and PC standards, while the triacylglycerol normalization factor curiously does not include the major influence of the TAG standard. This may be largely due to high variability of the TAG standard, with matrix-effects dominating the lipid class specific effects.

We also studied how the normalization by an internal standard for a specific functional lipid group would compare to normalization with the NOMIS method. We normalized the identified lysophosphatidylcholine, phosphatidylcholine, and triacylglycerol lipid species with the LPC, PC, and TAG standards, respectively. As shown in Table 2, the NOMIS method clearly outperforms the normalization of species from a specific molecular class based on appropriate internal standard.

**Table 2:** Comparison of coefficients of variance for three lipid classes The raw data variability for identified lipid species of the same class is compared to the results from the NOMIS method, as well as to results adjusted for an internal standard of the same class.

	Raw data	NOMIS	Internal standard
Lysophosphatidylcholines (N = 13)	0.245	0.094	0.221
Phosphatidylcholines (N = 74)	0.183	0.100	0.209
Triacylglycerols (N = 184)	0.227	0.146	0.308

### Normalization using *β *matrix obtained independently

The matrix **β **relates the variability of each individual metabolite with that of internal standards for a specific platform and biological matrix. Therefore, it is possible that the parameters **β **are obtained from a separate repeatability experiment involving large number of repeated measurements. This may often be desirable due to a large number of normalization parameters *N *x (*S *+ 2) determined by the method. The correction factors from Equation (17) in a real biological application then include the matrix **β **obtained independently and the measured levels of internal standards Ω_*sj *_from the biological experiment.

**Figure 8:**
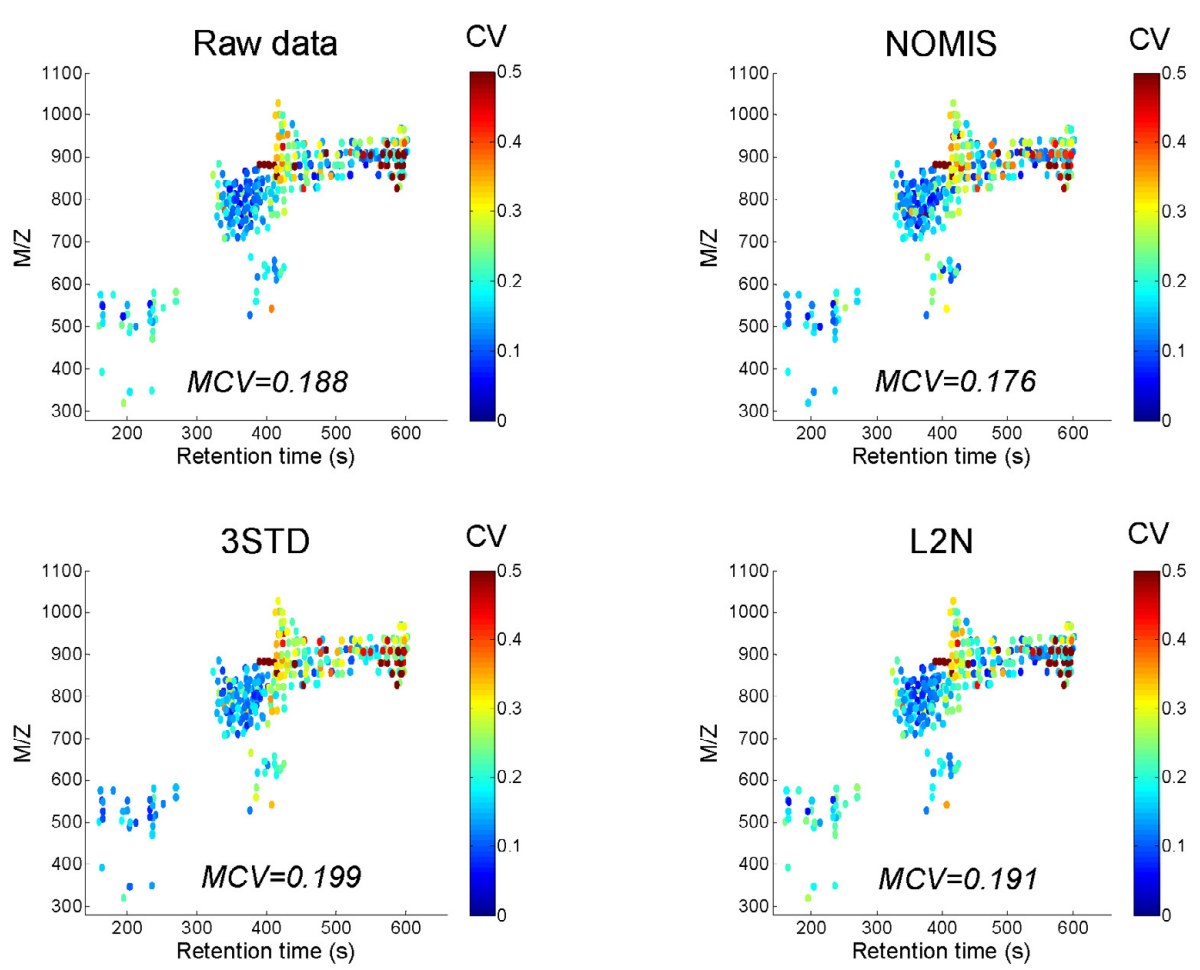
**Coefficients of variance for identified liver lipid species**. Each lipid molecular species is shown in the two dimensional plot of m/z *vs*. retention time plot, with the color corresponding to the coefficient of variance. The data is based on normalization performed on a different biological sample as in Figure 4, which was run 9 times (3 extractions, with 3 injections each). Total 360 identified lipid molecular species were included in the analysis. The NOMIS method utilized the **β **matrix calculated previously from a 16-sample run (Figure 4).

We tested this concept by utilizing the matrix **β **obtained from 360 identified lipid species in the above described 16 sample run experiment in a new experiment in repeatability conditions utilizing the same analytical platform but different biological sample (3 extracts, 3 injections each, i.e. total 9 sample runs). The results for the CVs for individual peaks are shown in Figure 8. Even in that case the NOMIS method outperformed the others, even though the differences as judged by median CV within each dataset were small.

These results provide evidence that the NOMIS method may also be used to develop a "reusable" **β **matrix for studies utilizing the same biological matrix and analytical platform. As evident from Equation

(16) (16) and demonstrated in Figure 7 for the case of lipids, the **β **matrix captures the variation of all detected metabolites in biological matrix as modeled by selected standard compounds. In case of repeatability runs demonstrated in this paper, the variability modeled is due to the *obscure *variation introduced in experimental studies of metabolites. We therefore believe the best usage of the NOMIS method should include a large run in repeatability conditions for a specific platform and specific biological matrix (i.e. biofluid or tissue type) in order to obtain a **β **matrix, which would then be applied to normalization of other samples using the same standard compounds and peak lists. The latter requires that the analytical method is sufficiently accurate and precise so that one can reliably track a specific set of peaks within a specific biological matrix even if some peaks remain unidentified. The **β **matrix may even be updated when new runs are made and we believe there are opportunities to develop sophisticated probabilistic methods to model and update the **β **matrix based on new experimental data.

The NOMIS normalization model is derived from the variabilities and correlation structure observed in data measured under repeatability conditions and does not specifically model different sources of systematic variation, incl. ion suppression. Therefore, as long as the assumption of multiplicative error model is valid to a reasonable extent, the NOMIS approach may be applicable. The heteroscedasticity of GC/MS spectra has in fact already been studied and demonstrated earlier [21]. We therefore believe the NOMIS approach may be applicable to analytical platforms other than LC/MS demonstrated in this paper.

## Conclusion

We introduced a new method for normalization of metabolomics data which utilizes variability information from multiple internal standard compounds to find optimal normalization factor for each individual molecular species detected (NOMIS). The method proved superior to two other commonly utilized normalization strategies in its ability to reduce variability across the full spectrum of metabolites.

The NOMIS method can be used directly as a one-step normalization method or as a two-step method where the normalization parameters containing information about the variabilities of internal standard compounds and their association to variabilities of metabolites are first calculated from a study carried in repeatability conditions. Additionally, the method can be used to select standard compounds for normalization and evaluate their influence on variability of all detected metabolites.

While we focused on applications of NOMIS to LC/MS based approaches; we believe the same strategy can be applied to other analytical platforms used in metabolomics, as well as to other levels of molecular profiling such as mass spectrometry based proteomics.

## Methods

### Liver LC/MS based lipid profiling

An aliquot of 20 μl of an internal standard mixture (5 reference compounds at concentration level 83–10 μg/ml), 50 μl of 0.15 M sodium chloride and chloroform: methanol (2:1) (200 μl) was added to the tissue sample (203–30 mg). The sample was homogenized, vortexed (2 min) let to stand (1 hour for liver) and centrifuged at 10000 RPM for 3 min. From the separated lower phase, an aliquot was mixed with 10 μl of a labelled standard mixture (3 stable isotope labelled reference compounds at concentration level 93–11 μg/ml) and 0.53–1.0 μl injection was used for LC/MS analysis.

Total lipid extracts were analysed on a Waters Q-Tof Premier mass spectrometer combined with an Acquity Ultra Performance LC™ (UPLC). The column, which was kept at 50°C, was an Acquity UPLCTM BEH C18 10 × 50 mm with 1.7 μm particles. The binary solvent system (flow rate 0.200 ml/min) included A. water (1% 1 M NH_4_Ac, 0.1% HCOOH) and B. LC/MS grade (Rathburn) acetonitrile/isopropanol (5:2, 1% 1 M NH_4_Ac, 0.1% HCOOH). The gradient started from 65% A/35% B, reached 100% B in 6 min and remained there for the next 7 min. The total run time per sample, including a 5 min re-equilibration step, was 18 min. The temperature of the sample organizer was set at 10°C.

Mass spectrometry was carried out on Q-Tof Premier (Waters, Inc.) run in ESI+ mode. The data was collected over the mass range of m/z 3003–1600 with a scan duration of 0.08 sec. The source temperature was set at 120°C and nitrogen was used as desolvation gas (800 L/h) at 250°C. The voltages of the sampling cone and capillary were 39 V and 3.2 kV respectively and collision energy 5 V, respectively. Reserpine (50 μg/L) was used as the lock spray reference compound (10 μl/min; 10 sec scan frequency).

Data processing including peak detection, alignment, and de-isotoping, was performed using the MZmine software version 0.60 [17]. Identification was performed based on an internal reference database of lipid species. The implementation of normalization methods and data analysis were performed using Matlab version 7.2 (Mathworks, Inc.).

## Abbreviations

MS: Mass spectrometry.

UPLC™: Ultra Performance Mass Spectrometry (Waters, Inc.).

LC/MS: Liquid chromatography – mass spectrometry.

GC/MS: Gas chromatography – mass spectrometry.

QTof: Quadrupole – time of flight mass spectrometer.

CV: Coefficient of variance.

MCV: Median coefficient of variance.

m/z: Mass-to-charge ratio (m is molecular mass and z is charge of the ion).

NOMIS: Normalization using Optimal selection of Multiple Internal Standards (the method introduced in this paper).

3STD: Normalization by retention-time-region-specific standard compounds.

L2N: Sum of squares normalization.

LPC: lysophosphatidylcholine

Cer: ceramide

PC: phosphatidylcholine

PE: phosphatidylethanolamine

TAG: triacylglycerol

## Authors' contributions

MSA participated in derivation of the method, data processing, and drafting of the manuscript. MK performed data analyses. LY performed processing and identification of lipid data. MO developed and derived the normalization method, performed data analyses, and drafted the manuscript. All authors read and approved the final manuscript.

## Supplementary Material

Additional file 1 Liver lipidomics dataset. The file includes data utilized in demonstration of the normalization method. Data was pre-processed with MZmine software version 0.60, using centroid peak detection, alignment, and de-isotoping steps. The column A shows whether the peaks correspond to internal standards (1) or metabolites (0). Columns B-D are self-descriptive. Columns from E on correspond to different sample runs and corresponding metabolite intensity levels (peak heights). The sample label is coded as < biological sample code>_REP < extraction replicate number> < injection replicate letter>. In the 16-sample repeatability run the sample B was utilized. Click here for file

## References

[B1] Raamsdonk LM, Teusink B, Broadhurst D, Zhang N, Hayes A, Walsh MC, Berden JA, Brindle KM, Kell DB, Rowland JJ, Westerhoff HV, van Dam K, Oliver SG (2001). A functional genomics strategy that uses metabolome data to reveal the phenotype of silent mutations. Nat Biotech.

[B2] Oresic M, Clish CB, Davidov EJ, Verheij E, Vogels JTWE, Havekes LM, Neumann E, Adourian A, Naylor S, Greef J, Plasterer T (2004). Phenotype characterization using integrated gene transcript, protein and metabolite profiling. Appl Bioinformatics.

[B3] Oresic M, Vidal-Puig A, Hanninen V (2006). Metabolomic approaches to phenotype characterization and applications to complex diseases. Expert Rev Mol Diagn.

[B4] Pauling L, Robinson AB, Teranishi R, Cary P (1971). Quantitative analysis of urine vapor and breath by gas-liquid partition chromatography. Proc Nat Acad Sci U S A.

[B5] van der Greef J, Davidov E, Verheij E, Vogels JTWE, van der Heijden R, Adourian AS, Oresic M, Marple EW, Naylor S, Harrigan GG and Goodacre R (2003). The role of metabolomics in systems biology: A new vision for drug discovery and development. Metabolic profiling: Its role in biomarker discovery and gene function analysis.

[B6] van der Greef J, Stroobant P, Heijden R (2004). The role of analytical sciences in medical systems biology. Curr Opin Chem Biol.

[B7] Goodacre R, Vaidyanathan S, Dunn WB, Harrigan GG, Kell DB (2004). Metabolomics by numbers: acquiring and understanding global metabolite data. Trends Biotechnol.

[B8] van den Berg RA, Hoefsloot HC, Westerhuis JA, Smilde AK, van der Werf MJ (2006). Centering, scaling, and transformations: improving the biological information content of metabolomics data. BMC Genomics.

[B9] de Hoffmann E, Stroobant V (2001). Mass spectrometry: Principles and applications.

[B10] Crawford LR, Morrison JD (1968). Computer methods in analytical mass spectrometry. Identification of an unknown compound in a catalog. Anal Chem.

[B11] Scholz M, Gatzek S, Sterling A, Fiehn O, Selbig J (2004). Metabolite fingerprinting: detecting biological features by independent component analysis. Bioinformatics.

[B12] Wang W, Zhou H, Lin H, Roy S, Shaler TA, Hill LR, Norton S, Kumar P, Anderle M, Becker CH (2003). Quantification of proteins and metabolites by mass spectrometry without isotopic labeling or spiked standards. Anal Chem.

[B13] Hartemink AJ, Gifford DK, Jaakkola TS, Young RA, Bittner M, Chen Y and Dorsel A (2001). Maximum likelihood estimation of optimal scaling factors for expression array normalization. Microarrays: optical technologies and informatics Proceedings of SPIE (vol 4266).

[B14] Bijlsma S, Bobeldijk I, Verheij ER, Ramaker R, Kochhar S, Macdonald IA, vanOmmen B, Smilde AK (2006). Large-scale human metabolomics studies: A strategy for data (pre-) processing and validation. Anal Chem.

[B15] Aitchison J (2003). The Statistical Analysis of Compositional Data.

[B16] Zhang Y, Proenca R, Maffei M, Barone M, Leopold L, Friedman JM (1994). Positional cloning of the mouse obese gene and its human homologue. Nature.

[B17] Katajamaa M, Oresic M (2005). Processing methods for differential analysis of LC/MS profile data. BMC Bioinformatics.

[B18] Steuer R, Kurths J, Fiehn O, Weckwerth W (2003). Observing and interpreting correlations in metabolomic networks. Bioinformatics.

[B19] Diggle P, Heagerty P, Liang KY, Zeger S (2002). Analysis of longitudinal data.

[B20] Katajamaa M, Miettinen J, Oresic M (2006). MZmine: toolbox for processing and visualization of mass spectrometry based molecular profile data. Bioinformatics.

[B21] Kvalheim OM, Brakstad F, Liang Y (1994). Preprocessing of analytical profiles in the presence of homoscedastic or heteroscedastic noise. Anal Chem.

[B22] Cleveland WS (1979). Robust locally weighted regression and smoothing scatterplots. J Am Stat Assoc.

